# Diversity and Distribution of Uncultured and Cultured Gaiellales and Rubrobacterales in South China Sea Sediments

**DOI:** 10.3389/fmicb.2021.657072

**Published:** 2021-06-16

**Authors:** Rou-Wen Chen, Yuan-Qiu He, Lin-Qing Cui, Cun Li, Song-Biao Shi, Li-Juan Long, Xin-Peng Tian

**Affiliations:** ^1^CAS Key Laboratory of Tropical Marine Bio-resources and Ecology, Guangdong Key Laboratory of Marine Materia Medica, RNAM Center for Marine Microbiology, South China Sea Institute of Oceanology, Chinese Academy of Sciences, Guangzhou, China; ^2^College of Earth and Planetary Sciences, University of Chinese Academy of Sciences, Beijing, China; ^3^Southern Marine Science and Engineering Guangdong Laboratory (Guangzhou), Guangzhou, China

**Keywords:** Actinobacteria, Gaiellales, Rubrobacterales, marine sediment, culture

## Abstract

Actinobacteria are ubiquitous in marine ecosystems, and they are regarded as an important, underexplored, potential pharmaceutical resource. The orders Gaiellales and Rubrobacterales are deep taxonomic lineages of the phylum Actinobacteria, both are represented by a single genus and contain only a few species. Although they have been detected frequently by high-throughput sequencing, their functions and characteristics in marine habitats remain unknown due to the lack of indigenous phenotypes. Here, we investigated the status of the orders in South China Sea (SCS) sediments using culture-independent and culture-dependent methods. Gaiellales is the second-most abundant order of Actinobacteria and was widely distributed in SCS sediments at water depths of 42–4,280 m, and four novel marine representatives in this group were successfully cultured. Rubrobacterales was present at low abundance in energy-limited marine habitats. An isolation strategy for Rubrobacterales from marine samples was proposed, and a total of 138 mesophilic Rubrobacterales strains were isolated under conditions of light and culture time combined with high-salinity or low-nutrient media. Marine representatives recovered in this study formed branches with a complex evolutionary history in the phylogenetic tree. Overall, the data indicate that both Gaiellales and Rubrobacterales can adapt to and survive in extreme deep-sea environments. This study lays the groundwork for further analysis of the distribution and diversity of the orders Gaiellales and Rubrobacterales in the ocean and provides a specific culture strategy for each group. The results open a window for further research on the ecological roles of the two orders in marine ecosystems.

## Introduction

The deep sea is a permanently dark, low-temperature, high hydrostatic pressure, nutrient-limited habitat that possesses great diversity in microbial organisms. The deep sea is considered a resource for mining new and potentially valuable microbial species (Hassan and Shaikh, 2017). Actinobacteria are an important component of the bacterial community and are widely distributed in marine environments (Lam, 2006; Ward and Bora, 2006). Since the discovery of novel bioactive compounds such as salinosporamides (Marizomib) from marine actinobacteria, searching for new or rare marine actinobacteria has become a focus of marine microbial research (Dhakal et al., 2017).

The orders Gaiellales and Rubrobacterales were established by Albuquerque et al. (2011) and Stackebrandt et al. (1997), respectively. They are two deep monogenetic branches in the phylogenetic tree of the phylum Actinobacteria (Salam et al., 2020), which is of considerable evolutionary importance. Members of the two orders are aerobic and chemoheterotrophic but are difficult to culture, severely restricting the study of their function. The order Gaiellales was proposed as a separate order from Rubrobacterales and contains only one species, *Gaiella occulta* (Albuquerque et al., 2011). The obligate cultivation strategy and insufficient data concerning cultivated species of Gaiellales has hindered its identification in marine ecosystems. Previous research revealed that Gaiellales was predominant in various extreme environments such as weathered serpentine rock (Khilyas et al., 2019), mangrove wetlands (Liu et al., 2019), saline–alkaline soil (Peng et al., 2017), wastewater treatment plants (Shu et al., 2015), and marine ascidians (Steinert et al., 2015). Rubrobacterales is an extremophilic actinobacteria that is abundant in sunlight-exposed biofilms and in the highly irradiated Chernobyl area (Ragon et al., 2011), and dominates in desiccated areas such as speleothems (Vardeh et al., 2018), the Atacama desert (Crits-Christoph et al., 2013), and arid soils (Bachar et al., 2012). The genus *Rubrobacter* is known for the first radiation-resistant species *Rubrobacter radiotolerans*, which was isolated from a radioactive spring (Yoshinaka et al., 1973; Suzuki et al., 1988). *Rubrobacter* species from terrestrial habitats are a potential source of bioactive compounds with ecological applications such as radiation-resistant, desiccant-resistant, and enzymatic radical scavengers (Albuquerque et al., 2014), but they are rarely isolated from marine habitats due to their slow growth and the difficulty of recovery (Kämpfer et al., 2014; Chen et al., 2018).

The limitation of detecting unculturable taxa in marine bacterial communities has been partially conquered via the application of molecular ecological technology (Daae et al., 2013). Most indigenous marine bacteria play key roles in the marine ecosystem but have not been cultured due to insufficient understanding of their physiology and environmental interactions (Vartoukian et al., 2010; Stewart, 2012). Hence, endeavors to devise specific cultivation strategies are important. It is widely accepted that culture-dependent and culture-independent surveys yield different insights into actinobacterial diversity (Vaz-Moreira et al., 2011; Maldonado et al., 2018). Both molecular-based and cultivation-based approaches have been applied in exploring the broad diversity of the obligate marine actinomycete genus *Salinispora* from marine sediments (Mincer et al., 2005). Combining these two approaches to describe actinobacterial diversity is feasible, as they are complementary and partly compensate each other’s inherent limitations (Sun et al., 2010).

In this study, we used culture-independent and culture-dependent analyses to examine the distribution, abundance, diversity, and evolutionary status of the two less-studied actinobacterial orders Gaiellales and Rubrobacterales in the South China Sea (SCS). Our study provides a specific culture strategy for each group. Using this method, we obtained hundreds of pure strains within these two groups. We also demonstrate that there are many potentially new taxa in the orders Gaiellales and Rubrobacterales in marine sedimentary environments.

## Materials and Methods

### Sediment Samples

**FIGURE 1:**
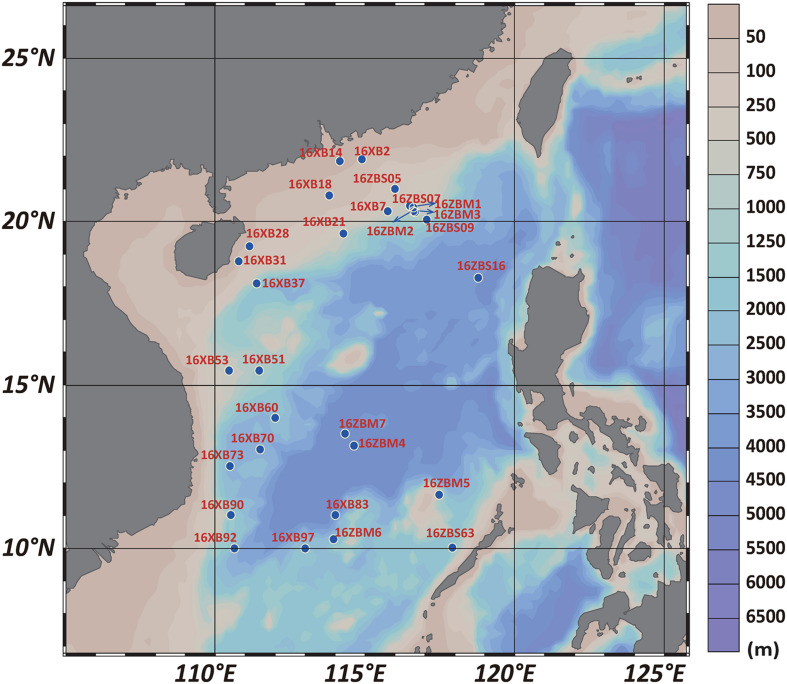
Map of sampling site locations in the SCS area.

Sampling was conducted during two open cruises in the SCS by R/V Shiyan 1 and R/V Shiyan 3. Twenty-nine sediment samples distributed over the SCS area from depths ranging from 42 to 4,280 m were collected from the sites shown in Figure 1. The name and site location information of samples are listed in Supplementary Table 1. A surface layer of sandy mud from 0 to 1 cm was obtained as a subsample aseptically after collecting using a grab-bucket collection sampler. The 29 sediment samples of the SCS were prepared for culture-independent experiments and stored at −20°C without pretreatment. All sediment samples were transported to the laboratory for further culture-dependent experiments.

### DNA Extraction, PCR Amplification, and Illumina Hiseq Sequencing

Total environmental DNA of the marine sediments was extracted using a DNeasy Power Soil Kit (MoBio, United States) following the manufacturer’s instructions. The V4 hypervariable region, about 400 bp of bacterial 16S rDNA, was amplified with prokaryotic universal primers 515F (5′-GTGCCAGCMGCCGCGGTAA-3′) and 806R (5′-GGACTACVSGGGTATCTAAT-3′) (Caporaso et al., 2011). PCR amplification was performed using TaKaRa Premix Taq version 2.0 (TaKaRa Biotechnology Co., Dalian, China) in a mixture with a final volume of 50 μl that contained 60 ng of DNA as a template, 10 μM of each primer, 25 μl of 2×Premix Taq, and nuclease-free water. The amplification was carried out using a BioRad S1000 (Bio-Rad Laboratory, CA, United States) thermocycler using the following procedure: 94°C for 5 min, 30 cycles of denaturation at 94°C for 30 s, primer annealing at 52°C for 30 s, and extension at 72°C for 30 s followed by a final extension at 72°C for 10 min and holding at 4°C. Each genomic DNA sample was amplified in triplicate. The quality of the purified PCR products was detected by 1% agarose gel electrophoresis. The PCR products for each sample were between 290 and 310 bp after concentration using GeneTools analysis software (Version 4.03.05.0, SynGene) (Beisvag et al., 2006). The required volume of PCR product for each replicate was calculated for each sample in accordance with the principle of equal quality. The mixture was recovered by an EZNA Gel Extraction Kit (Omega, United States) for further analysis. The amplicon library of purified PCR products of each sample was prepared using a NEBNext Ultra DNA Library Prep Kit (New England Biolabs, United States) following standard procedures. The construction of an amplification library of paired-end sequences was carried out on an Illumina Hiseq 2500 platform for PE250 sequencing according to the standard protocol (Guangdong Magigene Biotechnology Co., Ltd. Guangzhou, China).

### Sequence Processing and Analyzing

Raw reads of Illumina data were quality filtered by Trimmomatic (V0.33) (Bolger et al., 2014). Paired-end clean reads were retrieved after barcodes and primers were removed by Mothur software (V1.35.1) (Schloss et al., 2009). Paired-end clean reads were then concatenated by FLASH (V1.2.11) (Magoc and Salzberg, 2011). The sequences assigned to operational taxonomic units (OTUs) were clustered at the level of 97% sequence similarity by USEARCH (V8.0.1517) (Edgar, 2010). OTUs annotation was performed using QIIME against the Greengenes database (V13_5) (DeSantis et al., 2006). The rarefaction curves were calculated using QIIME packages (Caporaso et al., 2010), and the values of Shannon, Simpson, and Margalef indices were calculated by PRIMER 6 (Clarke and Gorley, 2006). Maps of the distribution and abundance of uncultured phenotypes in the SCS were generated by Ocean Data View (Schlitzer, 2002).

### Cultivation and Bacterial Taxonomic Classification

Samples were diluted two-fold with sterile seawater and mixed by vortexing before a 200 μl suspension was evenly spread on solid medium. Duplicated plates instead of triplicated plates of each medium were prepared for each sediment sample. Various types of separation media were designed and combined in the cultivation experiment for different nutritional requirements of the bacteria (Supplementary Table 2). Actinomycete Isolation Agar (AIA) medium, Marine Agar (MA)/MA-Starch media, and R2A medium were prepared with concentration gradients of various nutrients. The concentration of each medium was diluted to half, one-fifth, and one-tenth with water. Considering that some *Rubrobacter* species were reported to be moderately salt tolerant (Albuquerque et al., 2014), 10% (w/v) NaCl was added in some optimized media. Complex media and synthetic media were chosen and optimized by referring to published media for rare Actinobacteria, e.g., Acidimicrobiia, Thermoleophilia, and Rubrobacteria (Cleaver et al., 2007; Matsumoto et al., 2009; Matsumoto et al., 2013; He et al., 2020). The cultivation strategies were performed at different temperatures of 25–28, 37, and 55°C in petri dishes of two sizes (90 and 150 mm) and in the dark or under incandescent illumination of 8–12 μmol E m^–2^ s^–1^ at different time settings of 2–3 days, 7–14 days, and 1–3 months.

All colonies were selected to subculture on Marine Agar 2216E (MA, BD Difco^TM^) medium. At least three rounds of subculturing were performed to obtain pure cultures. Purified isolates were maintained in glycerol suspensions (20%, w/v) at −80°C. Genomic DNA of isolates was extracted by 100 μl of a reagent consisting of 5% (w/v) chelax-100 resin dissolved with distilled water as described by Walsh et al. (1991). PCR amplification of the 16S rRNA gene was performed with bacterial general primers 27F (5′-GAGTTTGATCCTGGCTCAG-3′) and 1492R (5′-GGTTACCTTGTTACGACTT-3′) as described by Rainey et al. (1996). The 16S rRNA gene sequence was assembled via the SeqMan program (version 7.1.0), and low-quality sequences were removed by the BioEdit program (Kimura, 1980). The nearly complete 16S rRNA gene sequence similarity analysis was carried out using EzBioCloud.^1^

### Phylogenetic Analysis

The OTU sequences from the 16S rRNA gene amplicon sequencing and the nearly complete 16S rRNA gene sequences from cultured strains were selected for phylogenetic reconstruction. The reference representatives were retrieved from the GenBank database. Phylogenetic analysis was calculated using the Kimura two-parameter model, and the cladogram was constructed by neighbor joining (Saitou and Nei, 1987) in the MEGA X software (Kumar et al., 2018) and visualized by the interactive Tree of Life (iTOL v4) tool (Letunic and Bork, 2019).

### PCA Analysis

Principal component analysis (PCA) allowed us to summarize and visualize the information concerning strains cultivated using multiple factors, including light intensity, culture time, salinity, dish size, temperature, and nutrient concentration. The cultured strains were divided into four groups: Gaiellales, Rubrobacterales, Actinomycetales, and non-Actinobacteria. The data were standardized automatically by the function PCA in FactoMineR (Le et al., 2008). The results provided a list of matrices (coordinates, correlations between variables and axes, squared cosine and sine contributions) for the variables that were extracted using the function get_pca_var, and similar results for the cultured strains were extracted using the function get_pca_ind from the PCA output in the factoextra R package (Kassambara and Mundt, 2020). The function fviz_pca_biplot in the factoextra R package was used to construct a biplot of cultured strains and variables (Kassambara and Mundt, 2020).

## Results

### Illumina Hiseq Sequencing Information

In this study, a total of 785,421 high-quality effective sequences were gained from the sample libraries, and the average length of these overlapping paired-end sequences was about 374 bp. There were from 16,549 to 55,059 sequences of 2,170 to 6,821 OTUs obtained from the samples (Supplementary Table 3). The rarefaction curve tended to approach the saturation plateau, indicating that the sampling sizes were sufficient (Supplementary Figure 1). A total of 536,417 sequences of bacteria were obtained, with from 11,794 to 37,153 sequences distributed in 29 samples assigned to 16,825 OTUs (Supplementary Table 3). The number of OTUs ranged from 1,839 to 6,042, and 93 OTUs were shared among all samples, indicating that the bacteria were well distributed in different samples from various water depths.

### Taxa of Uncultured Actinobacteria

**FIGURE 2:**
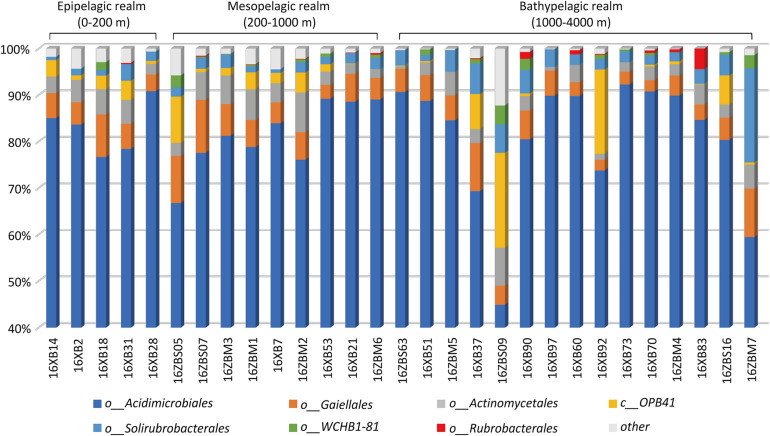
Actinobacterial composition in 29 deep-sea sediments.

A total of 22,074 sequences of 377 OTUs were assigned to the phylum Actinobacteria, and the average relative abundance of Actinobacteria was about 4.05%, ranging from 0.17 to 8.42%. The Shannon indices indicated that the actinobacterial diversity ranged from 2.77 to 6.25, while the Simpson indices ranged from 0.03 to 0.36. The Margalef indices indicated the actinobacterial species richness to be in the range 7.19 to 25.91 (Supplementary Table 3). Acidimicrobiales was the most abundant, accounting for 81.21% of Actinobacteria on average (Figure 2 and Supplementary Figure 2). The orders Gaiellales, Actinomycetales, and Solirubrobacterales were the next most dominant groups detected in all sediment samples in the SCS, accounting for 5.8, 3.81, and 3.34% of Actinobacteria on average, respectively. The remaining Actinobacteria were mainly from known or Candidatus orders, including group *OPB41*, group *WCHB1-81*, and Rubrobacterales (Supplementary Figure 2). The groups *OPB41* and *WCHB1-81* and the order Rubrobacterales were rare with low relative abundance in partial samples from the SCS (Figure 2).

### Taxa of Uncultured and Cultured Gaiellales and Rubrobacterales

**FIGURE 3:**
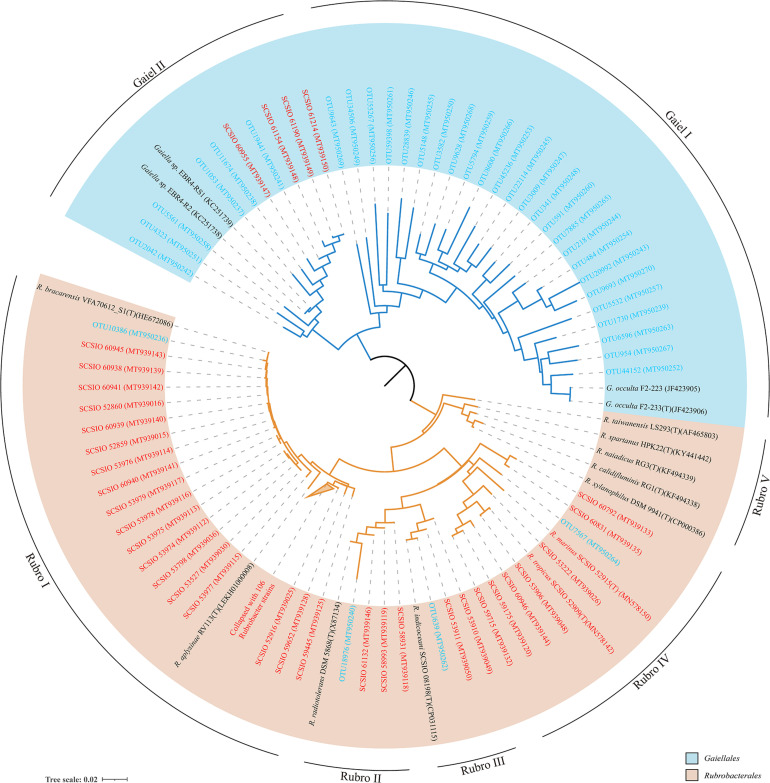
The neighbor-joining tree showing phylogenetic relationships of cultured and uncultured Gaiellales and Rubrobacterales based on nearly full-length 16S rRNA gene sequences (>1,300 bp) and OTUs using 374 unambiguous nucleotides. Tree bar, 0.02 sequence divergence. The similar *Rubrobacter* strains were collapsed with a 2% threshold of dissimilarity. Red font indicates isolated strains; blue font indicates the environmental OTUs. GenBank accession numbers used are given in parentheses.

There were 31 Gaiellales OTUs from 1,216 sequences detected in all sediment sample sites, and the relative abundance of the order was from 2.32 to 11.38% of the Actinobacteria (Supplementary Table 4). OTU 218 and OTU 484 were most frequently detected in the SCS, and OTUs 341 and 591 had the highest numbers in the 460 m depth environment sample (16ZBS07) (Supplementary Table 5). Thirty-one uncultured Gaiellales OTUs clustered with known Gaiellales representatives and formed multiple independent branches in the phylogenetic tree (Figure 3). Based on the culture-dependent method, only four Gaiellales strains were isolated from sediment samples taken at depths of 320–460 m, they were slow-growing on SN-Mn and MA plates and were better maintained in liquid MB media. They represented two potential new species by showing relatively low similarity (<89.0%) to the only known species *Gaiella occulta*. Four cultured strains were clustered with six uncultured OTUs and two unclassified species (*Gaiella* sp. EBR4-RS1 and *Gaiella* sp. EBR4-R2), they formed a separate cluster (named clade Gaiel II) far from the only known species *G. occulta* with a clear divergence and thus represent a new family group.

The order Rubrobacterales comprised a small proportion of Actinobacteria, with extremely low relative abundance (from 0.00 to 4.42% of Actinobacteria). The order was distributed sporadically in the SCS and occurred more in deep sea sediments than in the shallows. Four Rubrobacterales OTUs with 72 sequences were detected in nearly half of all samples, and two ubiquitous representatives, OTU 18976 and OTU 639, showed the highest numbers in the 3,503 m depth environmental sample (16XB83) (Supplementary Table 5). Finally, 138 strains assigned to the order Rubrobacterales were isolated from nine samples at depths from 323 to 4,280 m. Samples at water depths of 2,061, 3,448, and 460 m yielded 65, 34, and 21 *Rubrobacter* strains, respectively. The clustering tree of uncultured and cultured organisms revealed that they spanned five divergent phylogenetic lineages of the order Rubrobacterales (Figure 3). Hundreds of *Rubrobacter* strains and OTU 10386 were clustered together with two known species, *Rubrobacter aplysinae* and *Rubrobacter bracarensis*, as a part of clade Rubro I. Two sister groups, Rubro II and Rubro III, were ubiquitous in SCS sediments. Species *R. radiotolerans* and *R. indicoceani* were representative of the clades Rubro II and Rubro III, respectively; each had corresponding marine cultured strains and uncultured categories. Nine strains clustering with OTU 7567 formed a distinct phylogenetic cluster named clade Rubro IV and showed a great degree of novelty at the level of species diversity, with the highest similarity to other known species below 95%. Among these strains, we reported two strains as novel *Rubrobacter* species, *R. tropicus* and *R. marinus* (Chen et al., 2020). Moreover, the obtained 16S rRNAs of marine Rubrobacterales were most closely related to four species, *R. aplysinae*, *R. bracarensis*, *R. indicoceani*, and *R. radiotolerans.* However, no marine sequences in this study were related to Rubro V, which is composed of several terrestrial thermophilic species.

### The Cultivation of Gaiellales and Rubrobacterales

**FIGURE 4:**
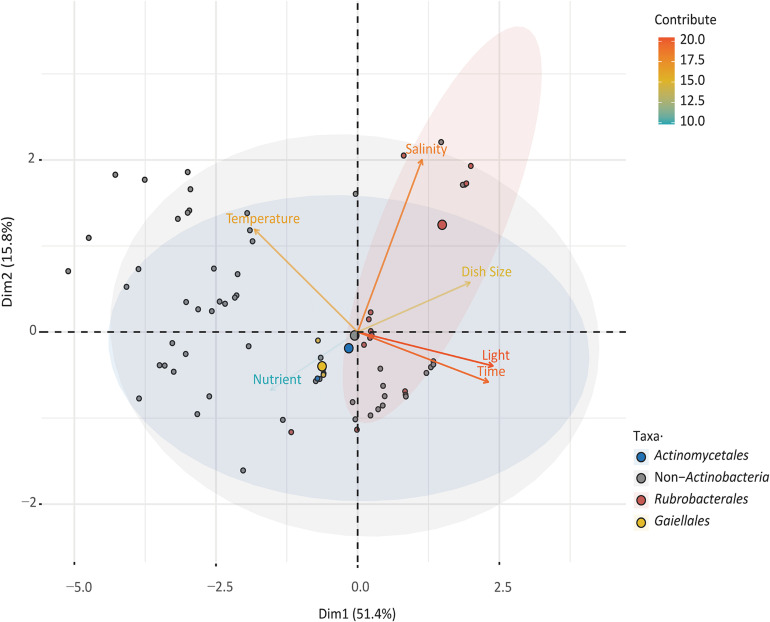
Principal component analysis (PCA) illustrating the relationship between culturable taxa with cultivation variables.

To explore the relationships between cultured species in the orders Gaiellales and Rubrobacterales and to compare the results with normal bacterial growth, six culture factors (culture time, salinity, dish size, temperature, and nutrient concentration) and 3,504 cultured strains were analyzed using PCA (Figure 4). The first two principal components explained 67.2% of the variance. The closer a variable is to the correlation ellipse, the better its representation on the factor map. In the biplot, the variables light, time, and salinity contributed the most to dimensions 1 and 2. Cultured Rubrobacterales strains yielded high values for four factors (light, culture time, salinity, and dish size), but had low values for variables temperature and nutrient concentration. The PCA results were consistent with the culture results. Under optimal light conditions, 109 *Rubrobacter* strains were cultured. The high-salinity (over 10% (w/v) NaCl) media (YJSF, AIAS, and CAAM) yielded the greatest number of halo-tolerant *Rubrobacter* isolates (Supplementary Figure 3). Although the oligotrophic media (AIAE, SN, and ZANT) obtained the second largest number of *Rubrobacter* isolates, marine Rubrobacterales isolates required sufficient nutrition for growth; this was shown by the addition of 1% glucose to the medium. In this study, Gaiellales and Rubrobacterales formed visible colonies on isolation media when the cultivation time extended to at least one month at 25–28°C incubation. Only four marine Gaiellales strains were isolated from the inorganic medium with manganese metal ions and MA medium; this result needs further research to find the root causes or bottlenecks for bacterial growth and to design additional cultivation strategies.

## Discussion

### Actinobacterial Vertical Distribution and Composition in the SCS

Actinobacteria are distributed globally in various marine habitats, including continental shelves, open ocean, and the deep sea (Ward and Bora, 2006; Jensen and Lauro, 2008). Previous studies have revealed the abundance of Actinobacteria in SCS sediment (relative abundance 4–10% of total sequences), which is more common in the deep sea than in the shallows (Zhu et al., 2013). In this study, the normally uneven fluctuation of actinobacterial abundance was generally stable in sediment environments (about 4.05%) (Supplementary Figure 4), but the diversity and species richness of Actinobacteria OTUs in shallow samples were higher than in deep samples (Supplementary Table 3). The composition of marine Actinobacteria at the level of order was remarkably similar to earlier research results (Durbin and Teske, 2011; Chen et al., 2016). The most abundant Actinobacteria group was Acidimicrobiales, and the next most dominant groups were the orders Gaiellales, Actinomycetales, and Solirubrobacterales while Rubrobacterales comprised a minor fraction of the Actinobacteria. The actinobacterial taxa composition difference in the samples may be related to niche adaptation for inhabiting marine environments.

### Distribution of Gaiellales and Rubrobacterales in the Deep Sea

Our study revealed that uncultured Gaiellales sequences were widely distributed from 42 to 4,280 m in SCS sediment environments, and Gaiellales was the second dominated uncultured marine Actinobacteria. Previous studies also showed that the order Gaiellales was predominant in a variety of marine habitats, such as submarine permafrost water, permafrost sediments (Mitzscherling et al., 2017), mangrove wetlands (Liu et al., 2019) and deep sea (Chen et al., 2016). The distribution patterns calculated by the diversity indices (Shannon, Simpson, and Margalef) showed that there was no obvious trend in the diversity of the two orders with the depth of sediment environments (Supplementary Figure 5). But the OTUs belonging to the clade Gaiel I were particularly abundant in the mesopelagic sediments (Supplementary Table 5) where the temperature ranged from about 8 to 2.5°C (Yang et al., 2018). Although it is unclear whether this high abundance is related to the adaptation to the middle and deep sea, this result further illustrated that Gaiellales is ubiquitous and widely distributed in SCS sediments, and also has a stronger ability to adapt to marine environment than the order Rubrobacterales.

The order Rubrobacterales was a ubiquitous group and had greater natural diversity, such as ancient vestiges (Schabereiter-Gurtner et al., 2001; Mihajlovski et al., 2017), rocky coasts (Molina-Menor et al., 2019), Arctic desert endostromatolites (Pellerin et al., 2009), etc. Rubrobacterales were also detected by high-throughput sequencing although the group comprised only a small proportion of the microorganisms in marine habitats (Brown and Bowman, 2001; Kochling et al., 2011). Similar results were also obtained in this study using the culture-independent method. But for the culture-dependent method, the optimal growth occurred when adding 1% glucose to the media, which implied that the bacteria may be restricted to deep sea sediments where nutrients are limited. They are actually found in deep sea sediments, where they were not detected by the uncultured method. The bacteria mainly exist in the bathypelagic sediments at depths of over 2,000 m, where the temperature is generally below 2.5°C (Yang et al., 2018). Therefore, we postulate that the order Rubrobacterales has cells with a slow growth disadvantage, and they may struggle to survive in nutritionally limited marine habitats.

### Diversity of Gaiellales and Rubrobacterales in the Deep Sea

Previous results from interspecies heterogeneity of 16S rRNA genes showed that Gaiellales had a more complex genetic evolutionary history than Rubrobacterales, and this order may have undergone more evolutionary events in the marine environment (Steinert et al., 2015). However, only one valid published species in the order Gaiellales is not enough to supply the representative Candidatus for classifying cultured-independent OTUs, which severely limits the study of the order’s diversity and phylogeny. New marine representative species and marine OTUs in this study were clustered into two separated groups named Gaiel I and Gaiel II, that represent potential new marine-derived higher-level taxa. The results support Gaiellales as having a large family tree with complex branches and a high level of evolution in the ocean, a hypothesis that is consistent with their wide distribution and high abundance in marine habitats.

For the order Rubrobacterales, previous study illustrated the limitations of analyzing its diversity and phylogeny by culture-independent surveys, due to the low abundance in marine environments (Rappe et al., 1999; Yooseph et al., 2007; Sunagawa et al., 2015). The deeper recognition of the special niche of rare uncultured Rubrobacterales in marine habitats required more pure cultured strains or genus-specific primers (Castro et al., 2019). In this study, four OTUs and 138 cultured strains clustered in four groups were found to be associated with oceans, expanding the phylogenetic tree of Rubrobacterales. And clade Rubro IV was clearly novel, as shown by the independence and the marine-derived characteristics. The phylogenetic tree of Rubrobacterales also showed that the evolution of these diverse strains is continuous in the marine ecosystem, a conjecture that perhaps they survived in the ancient, stable oceans and developed unique marine properties to adapt to the extreme deep-sea environments.

### Isolation Strategies for Rubrobacterales and Gaiellales

It is necessary to successfully cultivate rare living species through a culture strategy, especially for the communities that are common but have low abundance. These rare species may serve as a potentially inexhaustible reservoir of genomic innovation, a factor that could explain how microbial communities episodically reshape planetary processes (Sogin et al., 2006). To obtain the optimal growth conditions for marine Rubrobacterales, an isolating strategy to improve the survival capacity in the laboratory was designed by simulating their natural environment to reverse the situation of nutritional disadvantage.

The *Rubrobacter* species survive well under illuminated conditions, with the ability to respond to reactive oxidative stress (ROS) and to efficiently repair DNA lesions (Egas et al., 2014). It has been reported that the growth of xero-tolerant heterotrophic *Rubrobacter* spp. can be promoted by decreased humidity and increased temperature combined with enhanced daylight irradiation (Imperi et al., 2007). In this study, compared to other light-sensitive bacteria that live in the dark aphotic zone of the deep ocean, the viability and competitive advantage of cultured Rubrobacterales on plates were enhanced by light, and 109 *Rubrobacter* strains were successfully isolated. Although most *Rubrobacter* species derived from terrestrial habitats are thermophilic (Carreto et al., 1996; Chen et al., 2004; Albuquerque et al., 2014; Norman et al., 2017), no isolates of the order Rubrobacterales showed thermotolerant ability in this study. Instead, 44 *Rubrobacter* strains were isolated from Indian Ocean sediments by incubation at 4°C for one year. Since marine sediments are largely present in low-temperature environments, the presence of low temperature-adapted bacteria with low thermostable enzymes (inactivated at temperatures over 40°C) would be expected (Hardeman and Sjoling, 2007). This is consistent with the result that no growth was observed at temperatures over 40°C in marine *Rubrobacter* species (Kämpfer et al., 2014; Chen et al., 2018).

When designing the synthetic media, the salinity and nutrient levels were first considered. In the present study the greatest numbers of halo-tolerant *Rubrobacter* isolates were obtained from high-salinity media, and the second-highest quantity was obtained using oligotrophic media. We speculate that the high-salinity or oligotrophic media restricted the growth of fast-growing bacteria, and the eutrophic bacteria were intolerant to starvation and ultimately died (Gray et al., 2019). Marine *Rubrobacter* species grew slowly and took a long time to form red colonies on plates (Kämpfer et al., 2014; Chen et al., 2018). Similarly, marine Gaiellales and Rubrobacterales were successfully cultured when the cultivation time was extended to at least one month. The subcultures also required at least two weeks to form rich visible colonies on plates. Recent results show that slow-growing colonies are usually discovered at a nutritional disadvantage status when one fast-growing strain is competing for nutrition resources under the same culture conditions (Carini, 2019). Hence, prolonging the incubation time can increase the ratio of viable counts of rare, slow-growing bacteria on media (Stevenson et al., 2004). Moreover, a drastic increase in the quantity of *Rubrobacter* colonies was discovered in this study using large-size petri dishes (150 mm), perhaps due to competition for the living space and the reduced competitive pressure for slowly growing or poorly adaptable bacteria such as Rubrobacterales and Gaiellales.

For the order Gaiellales, the sole known representative was isolated from a mineral water bottling plant, where the borehole water had a temperature of 28°C, a pH of 5.9, and was poor in mineral ions (Albuquerque et al., 2011). However, the lack of phenotypic and genomic annotated information increased the difficulty of the culture (Severino et al., 2019). One interesting result is that Gaiellales are strict chemoorganotrophs, as inferred by genomic data, but they could be cultured from an inorganic medium (SN-Mn) made up of 50% seawater in this study. This suggests that their growth may depend on certain nutrients in seawater that are not present in the laboratory and thereby make them difficult to culture. Further results showed that marine Gaiellales strains were hygrophilous and halophilic, different from the species *G. occulta*. Therefore, simulating the natural environment, especially its potential key factors, is an effective strategy for isolating novel, rare, or uncultured bacteria (Mu et al., 2020).

## Conclusion

In this study, we analyzed the status of the orders Gaiellales and Rubrobacterales in marine sediments of the SCS using culture-independent and culture-dependent methods. We concluded the following: (1) The order Gaiellales was the second-most dominant order of Actinobacteria, distributed in all detected sediment samples in different water depths of the SCS, but it could hardly be cultured; the order Rubrobacterales was present in low abundance but displayed a steady existence in over more than half of the marine sediments. (2) Marine Gaiellales are highly diverse in the ocean, and they can be separated into two main branches as higher-level new taxa. Each branch was represented by cultured representatives; marine Rubrobacterales clustered in four groups were associated with four known species, *R. aplysinae*, *R. bracarensis*, *R. indicoceani*, and *R. radiotolerans*. Clade Rubro IV is a novel independent branch from the deep sea. (3) Light, high salinity, culture time, or low nutrient levels at optimal growth temperature were the most effective factors for Rubrobacterales survival under laboratory conditions. The order Gaiellales may depend on certain marine factors for growth, and their ability to be cultured needs to be researched further by mimicking the natural habitat.

## Data Availability Statement

The datasets presented in this study can be found in the NCBI database with accession numbers SRR12534320–SRR12534348 and MT939011–MT950270.

## Author Contributions

X-PT and L-JL designed the workflow. R-WC and Y-QH collected the environmental samples and performed the cultivation and also prepared the manuscript. CL and L-QC helped in data collection and making figure and tables. S-BS helped in improving the manuscript. All authors reviewed and corrected the manuscript.

## Conflict of Interest

The authors declare that the research was conducted in the absence of any commercial or financial relationships that could be construed as a potential conflict of interest.

## Footnotes

1 https://www.ezbiocloud.net/identify
