# Supplementary material for: Diversity and Distribution of Uncultured and Cultured Gaiellales and Rubrobacterales in South China Sea Sediments
Source: Front Microbiol. 2021 Jun 16;12:657072. doi: 10.3389/fmicb.2021.657072 (PMC8248818; doi:10.3389/fmicb.2021.657072)
Supplement: Supplementary file 1 [file Table_1.docx]

**Supplementary Table 1.** Information for the sediment samples were collected from the South China Sea

| **No.** | **Sample ID** | **Longitude (^o^E)** | **Latitude (^o^N)** | **Water depths (m)** | **Sampling date** | **Sampling ship** |
| --- | --- | --- | --- | --- | --- | --- |
| 1 | 16XB14 | 114.184 | 21.850 | 42 | 2016.09.16 | R/V Shiyan 3 |
| 2 | 16XB2 | 114.909 | 21.904 | 67 | 2016.09.16 | R/V Shiyan 3 |
| 3 | 16XB18 | 113.805 | 20.805 | 88 | 2016.09.11 | R/V Shiyan 3 |
| 4 | 16XB31 | 110.796 | 18.791 | 96 | 2016.09.19 | R/V Shiyan 3 |
| 5 | 16XB28 | 111.154 | 19.252 | 99 | 2016.09.19 | R/V Shiyan 3 |
| 6 | 16ZBS05 | 115.998 | 21.006 | 323 | 2016.09.04 | R/V Shiyan 1 |
| 7 | 16ZBS07 | 116.502 | 20.505 | 460 | 2016.09.05 | R/V Shiyan 1 |
| 8 | 16ZBM3 | 116.700 | 20.287 | 596.1 | 2016.09.05 | R/V Shiyan 1 |
| 9 | 16ZBM1 | 116.652 | 20.449 | 610.7 | 2016.09.05 | R/V Shiyan 1 |
| 10 | 16XB7 | 115.764 | 20.311 | 630 | 2016.09.17 | R/V Shiyan 3 |
| 11 | 16ZBM2 | 116.635 | 20.311 | 654.5 | 2016.09.05 | R/V Shiyan 1 |
| 12 | 16XB53 | 110.462 | 15.449 | 711 | 2016.09.25 | R/V Shiyan 3 |
| 13 | 16XB21 | 115.280 | 19.631 | 855 | 2016.09.11 | R/V Shiyan 3 |
| 14 | 16ZBM6 | 113.981 | 10.289 | 933 | 2016.09.17 | R/V Shiyan 1 |
| 15 | 16ZBS63 | 117.935 | 10.023 | 1102 | 2016.09.19 | R/V Shiyan 1 |
| 16 | 16XB51 | 111.494 | 15.456 | 1293 | 2016.09.25 | R/V Shiyan 3 |
| 17 | 16ZBM5 | 117.502 | 11.656 | 1684 | 2016.09.20 | R/V Shiyan 1 |
| 18 | 16XB37 | 111.399 | 18.123 | 1737 | 2016.09.20 | R/V Shiyan 3 |
| 19 | 16ZBS09 | 117.062 | 20.077 | 1781 | 2016.09.05 | R/V Shiyan 1 |
| 20 | 16XB90 | 110.524 | 11.012 | 1937 | 2016.10.04 | R/V Shiyan 3 |
| 21 | 16XB97 | 113.013 | 10.012 | 2056 | 2016.10.05 | R/V Shiyan 3 |
| 22 | 16XB60 | 112.003 | 13.997 | 2061 | 2016.09.27 | R/V Shiyan 3 |
| 23 | 16XB92 | 110.639 | 10.013 | 2437 | 2016.10.04 | R/V Shiyan 3 |
| 24 | 16XB73 | 110.500 | 12.527 | 2528 | 2016.09.29 | R/V Shiyan 3 |
| 25 | 16XB70 | 111.503 | 13.050 | 2769 | 2016.09.29 | R/V Shiyan 3 |
| 26 | 16ZBM4 | 114.639 | 13.144 | 3448 | 2016.09.22 | R/V Shiyan 1 |
| 27 | 16XB83 | 114.018 | 11.032 | 3503 | 2016.10.02 | R/V Shiyan 3 |
| 28 | 16ZBS16 | 118.800 | 18.295 | 4000 | 2016.09.08 | R/V Shiyan 1 |
| 29 | 16ZBM7 | 114.356 | 13.510 | 4280 | 2016.09.22 | R/V Shiyan 1 |
