# Supplementary material for: Diversity and Distribution of Uncultured and Cultured Gaiellales and Rubrobacterales in South China Sea Sediments
Source: Front Microbiol. 2021 Jun 16;12:657072. doi: 10.3389/fmicb.2021.657072 (PMC8248818; doi:10.3389/fmicb.2021.657072)
Supplement: Supplementary file 2 [file Table_2.docx]

**Supplementary Table 2.** The genre and ingredients of different media

| **Category and composition of isolation medium (g/l)** |
| --- |
| **AIA type media** (Chen et al., 2018).**:**  **AIA** (Actinomycete Isolation Agar, BD Difco^TM^): Asparagine 0.1, Caseinate 2.0, Dipotassium Phosphate 0.5, Sodium Propionate 4.0, Ferrous Sulfate 1$\times$10^-3^, Magnesium Sulfate 0.1, Agar 15.0.  **AIAB**: 50% AIA powder, Agar up to 15.0.  ***AIAE**: 20% AIA powder, Agar up to 15.0.  ***AIAJ**: 10% AIA powder, Agar up to 15.0.  **MA type media** (Matsumoto et al., 2013):  ***MA** (Marine Agar 2216E, BD Difco^TM^)：Bacto Peptone 5.0, Bacto Yeast extract 1.0, Calcium Chloride 1.8, Ferric citrate 0.1, Potassium Bromide 0.08, Potassium Chloride 0.55, Boric Acid 0.02, Magnesium Chloride 5.90, Sodium Chloride 19.45, Sodium Carbonate 0.16, Sodium Fluoride 2.4$\times$10^-3^, Ammonium Nitrate 1.60 $\times$10^-3^, Disodium Hydrogen Phosphate 8$\times$10^-3^, Sodium Silicate 4$\times$10^-3^, Sodium Sulfate 3.24, Strontium Chloride 3.4$\times$10^-2^, Agar 15.0.  ***MAB**: 50% MA powder, Agar up to 15.0.  **MAE**: 20% MA powder, Agar up to 15.0.  **MAJ**: 10% MA powder, Agar up to 15.0.  **MA-Starch type media:**  **MABS**: MAB, 1% (w/v) Soluble Starch.  ***MAES**: MAE, 1% (w/v) Soluble Starch.  ***MAJS**: MAJ, 1% (w/v) Soluble Starch.  **MAS**: MA powder, 1% (w/v) Soluble Starch, Agar up to 15.0.  **MATS**: 5% MA powder, 1% (w/v) Soluble Starch, Agar up to 15.0.  **R2A type media** (Albuquerque et al., 2011)  ***R2A**: Yeast extract 0.5, Peptone 0.5, Casamino Acids 0.5, Glucose 0.5, Soluble Starch 0.5, Na-pyruvate 0.3, Dipotassium Phosphate 0.3, Magnesium Sulfate 0.05, Agar 15.00. (Final pH 7.2).  **R2AB**: 50% R2A powder, Agar up to 15.0.  **R2AJ**: 10% R2A powder, Agar up to 15.0.  ***R2AM**: R2A medium, Magnesium Chloride hexahydrate 10.0, Magnesium Sulfate heptahydrate 6.0, Calcium Chloride dihydrate 2.0, Potassium Chloride 0.6.  **High salinity media:**  ***AIAS**: AIA powder 10.0, Sodium Chloride 100.0, Sea salt 5.0, Strontium Chloride 2.0, Agar up to 15.0.  **BFSM**: Beef extract 2.0, Calcium Carbonate 1.0, Sea salt 5.0, [Sodium Molybdate](http://www.so.com/link?url=http://dict.youdao.com/search?q=%5b%E6%97%A0%E5%8C%96%5d%20sodium%20molybdate&keyfrom=hao360&q=%E9%92%BC%E9%85%B8%E9%92%A0+%E8%8B%B1%E8%AF%AD&ts=1513758001&t=8283e48c14dd14a71c946860f7060d6) 5.0, Soluble Starch 2.0, Sodium Chloride 100.0, Agar 15.0.  ***CAAM**: Hydrolysate Casein 1.0, Potassium Chloride 2.0, Magnesium Sulfate 2.0, Sodium Chloride 100.0, Sea salt 10.0, Sodium Glutamate 1.0, Trisodium Citrate 1.0, Yeast extract 1.0, Agar 15.0, Potassium Permanganate 2.0 (Autoclaved Alone)；  ***YJSF**: Marine Broth 2216E powder (MB, BD Difco^TM^) 15.0, Calcium Carbonate 5.0, Sodium Chloride 100.0, Agar 15.0, Ferric Chloride 0.5 (Filtration Autoclaved), Ferrous Sulfate 0.5 (Filtration Autoclaved).  **Complex media:**  **GMP**: Glucose 10.0, Peptone 5.0, Meat extract 5.0, Sodium Chloride 3.0, Agar 15.  **MYP**: Malt extract 5.0, Yeast extract 5.0, Peptone 5.0, Sodium Chloride 3.0, Agar 15.0.  **PDA** (Potato Dextrose Agar, BD Difco^TM^)：Potato Starch 4.0, Dextrose 20.0, Agar 15.0.  **TM**: Yeast extract 1.0, Tryptone 1.0, Nitrilotriacetic acid 0.1, Calcium Sulfate 0.04, Magnesium Chloride 0.2, 0.01M Ferric Citrate 0.50 ml, Trace element solution (see as follows) 0.50 ml, Phosphate buffer (see as follows) 100.00 ml, Agar 15.0, (Final pH 7.2). Trace element solution (g/l): Sulfuric Acid 0.5 ml, Manganese Sulfate 2.3, Zinc Sulfate 0.5, Boric Acid 0.5, Copper Sulfate 0.025, Sodium Molybdate 0.025, Cobalt Chloride 0.45. Phosphate buffer (g/l): Monopotassium Phosphate 5.4, Disodium Hydrogen Phosphate 43.0.  **TSA** (Tryptone Soya Agar, BD Difco^TM^) (Kämpfer et al., 2014): Casein 17.0, Soymeal Peptone 3.0, D-Glucose 2.5, Sodium Chloride 5.0, Dipotassium Phosphate 2.5, Agar 15.0, (Final pH 7.2).  ***R** (Matsumoto et al., 2009): peptone 10.0, Yeast extract 5.0, Malt extract 5.0, Casamino Acids 5.0, Beef extract 2.0, Glycerol 2.0, Tween 80 0.05, Magnesium Sulfate 1.0, Agar 15.0.  **Synthetic media:**  **AM** (*Acidimicrobium* Medium) (Cleaver et al., 2007): Magnesium Sulfate 0.5, Ammonium Sulfate 0.4, Dipotassium Phosphate 0.2, Potassium Chloride 0.1, Ferric Sulfate 0.01, Yeast extract 0.25, Agar 15.0.  ***AMC**: AM medium and casamino acid 0.25  **GN1** (GAUZE’s Medium): Soluble Starch 20.0, Potassium Nitrate 1.0, Sodium Chloride 0.5, Magnesium Sulfate 0.5, Dipotassium Phosphate 0.5, Ferric Sulfate 0.01, Agar 15.0.  ***SN** (He et al., 2020): Sodium Nitrate 0.75, Dipotassium Phosphate 0.0159, Disodium EDTA dihydrate 0.0056, Sodium Carbonate 0.0104, 50% Seawater, Agar 15.0, Vitamin B12 0.001g (Filtration Autoclaved), Cyano trace metal solution 1$\times$10^-3^ (Citric Acid 6.25, Ferric Ammonium Citrate 6.0, Manganese Chloride 1.4, Sodium Molybdate 0.39, Cobalt Nitrate 0.025, Zinc Sulfate 0.222, ultrapure water 1000 ml).  ***SN-Mn**: SN medium, 10X Manganese Chloride  ***ZANT:** Sodium Bicarbonate 2.0, Sodium Dihydrogen Phosphate 0.05, Sodium Nitrate 0.5, Calcium Chloride 0.02, Magnesium Sulfate 0.05, Potassium Chloride 0.1, A5 1×10-3 (Boric Acid 2.86, Manganese Chloride 1.80, Zinc Sulfate 0.22, Sodium Molybdate 0.3, Copper Sulfate 0.08, ultrapure water 1000 ml) |

Note: * indicates the medium that *Rubrobacter* or *Gaiella* strains were isolated.
