# Supplementary material for: Diversity and Distribution of Uncultured and Cultured Gaiellales and Rubrobacterales in South China Sea Sediments
Source: Front Microbiol. 2021 Jun 16;12:657072. doi: 10.3389/fmicb.2021.657072 (PMC8248818; doi:10.3389/fmicb.2021.657072)
Supplement: Supplementary file 3 [file Table_3.docx]

**Supplementary Table 3.** General sequencing data from the sample libraries.

| **Sample ID** | **No. of Seqs^W^** | **No. of OTUs^W^** | **No. of Seqs^B^** | **No. of OTUs^B^** | **No. of Seqs^A^** | **No. of OTUs^A^** | **Ra^A^%** | **Shannon^A^** | **Simpson^A^** | **Margalef^A^** |
| --- | --- | --- | --- | --- | --- | --- | --- | --- | --- | --- |
| 16XB14 | 19250 | 3482 | 11794 | 3124 | 281 | 88 | 2.38 | 5.69 | 0.03 | 15.43 |
| 16XB2 | 16999 | 3406 | 12145 | 3064 | 208 | 73 | 1.71 | 5.56 | 0.04 | 13.49 |
| 16XB18 | 18376 | 3717 | 13910 | 3234 | 240 | 84 | 1.73 | 5.80 | 0.03 | 15.14 |
| 16XB31 | 18907 | 3624 | 13094 | 3244 | 389 | 102 | 2.97 | 5.91 | 0.03 | 16.94 |
| 16XB28 | 21889 | 3261 | 16617 | 2942 | 786 | 74 | 4.73 | 4.13 | 0.15 | 10.95 |
| 16ZBS05 | 16549 | 3325 | 14125 | 3000 | 502 | 99 | 3.55 | 5.87 | 0.02 | 15.76 |
| 16ZBS07 | 44962 | 6056 | 35725 | 5335 | 1564 | 173 | 4.38 | 5.72 | 0.04 | 23.39 |
| 16ZBM3 | 39695 | 5098 | 28661 | 4589 | 1241 | 154 | 4.33 | 5.68 | 0.04 | 21.48 |
| 16ZBM1 | 40541 | 5739 | 24424 | 4928 | 1152 | 168 | 4.72 | 6.18 | 0.03 | 23.69 |
| 16XB7 | 17893 | 3312 | 14712 | 2996 | 268 | 83 | 1.82 | 5.65 | 0.03 | 14.67 |
| 16ZBM2 | 55059 | 6821 | 37153 | 6042 | 1719 | 194 | 4.63 | 6.25 | 0.03 | 25.91 |
| 16XB53 | 20086 | 3322 | 13053 | 2918 | 564 | 73 | 4.32 | 3.89 | 0.19 | 11.37 |
| 16XB21 | 18190 | 2978 | 12933 | 2801 | 938 | 102 | 7.25 | 4.74 | 0.10 | 14.76 |
| 16ZBM6 | 23532 | 3297 | 19896 | 2817 | 930 | 67 | 4.67 | 4.07 | 0.15 | 9.66 |
| 16ZBS63 | 34070 | 3829 | 22443 | 3481 | 1231 | 74 | 5.49 | 3.71 | 0.22 | 10.26 |
| 16XB51 | 18717 | 3613 | 14995 | 3032 | 595 | 58 | 3.97 | 3.12 | 0.32 | 8.92 |
| 16ZBM5 | 47046 | 4582 | 32310 | 4167 | 1399 | 121 | 4.33 | 4.50 | 0.12 | 16.57 |
| 16XB37 | 22638 | 4851 | 16477 | 4119 | 531 | 120 | 3.22 | 5.79 | 0.04 | 18.96 |
| 16ZBS09 | 34482 | 2583 | 28426 | 2125 | 49 | 29 | 0.17 | 4.61 | 0.05 | 7.19 |
| 16XB90 | 21453 | 3672 | 16301 | 3117 | 548 | 67 | 3.36 | 3.79 | 0.18 | 10.47 |
| 16XB97 | 21854 | 2874 | 13431 | 2547 | 758 | 61 | 5.64 | 3.46 | 0.26 | 9.05 |
| 16XB60 | 26892 | 2919 | 12708 | 2585 | 568 | 54 | 4.47 | 3.16 | 0.30 | 8.36 |
| 16XB92 | 27342 | 3843 | 20496 | 3220 | 1726 | 93 | 8.42 | 3.63 | 0.18 | 12.34 |
| 16XB73 | 24868 | 3022 | 13839 | 2587 | 543 | 55 | 3.92 | 2.97 | 0.31 | 8.58 |
| 16XB70 | 25905 | 3456 | 15597 | 2952 | 749 | 58 | 4.80 | 2.89 | 0.34 | 8.61 |
| 16ZBM4 | 32357 | 3401 | 17746 | 2917 | 653 | 51 | 3.68 | 2.77 | 0.36 | 7.71 |
| 16XB83 | 27157 | 3354 | 14691 | 2954 | 815 | 71 | 5.55 | 3.94 | 0.19 | 10.44 |
| 16ZBS16 | 25871 | 3184 | 16730 | 2878 | 915 | 88 | 5.47 | 4.20 | 0.12 | 12.76 |
| 16ZBM7 | 22841 | 2170 | 11985 | 1839 | 212 | 45 | 1.77 | 4.43 | 0.08 | 8.21 |

*^W^ Total clean reads after pooling of samples according to the Qiime pipeline.*

*^B^ Bacteria.*

*^A^ Actinobacteria.*

*Ra^A^% The relative abundance of Actinobacteria.*

*Shannon ^A^, Simpson^A^, and Margalef^A^ indices were calculated after subsampling of 22,074 reads for Actinobacterial samples.*
