# Supplementary material for: Diversity and Distribution of Uncultured and Cultured Gaiellales and Rubrobacterales in South China Sea Sediments
Source: Front Microbiol. 2021 Jun 16;12:657072. doi: 10.3389/fmicb.2021.657072 (PMC8248818; doi:10.3389/fmicb.2021.657072)
Supplement: Supplementary file 4 [file Table_4.docx]

**Supplementary Table 4. S**equence analysis data of the orders *Gaiellales* and *Rubrobacterales* from the sample libraries.

| **Sample ID** | **No. of Seqs^G^** | **No. of OTUs^G^** | **Ra^G^%** | **No. of Seqs^R^** | **No. of OTUs^R^** | **Ra^R^%** |
| --- | --- | --- | --- | --- | --- | --- |
| 16XB14 | 15 | 6 | 5.34 | 0 | 0 | 0.00 |
| 16XB2 | 10 | 6 | 4.81 | 0 | 0 | 0.00 |
| 16XB18 | 22 | 6 | 9.17 | 0 | 0 | 0.00 |
| 16XB31 | 21 | 5 | 5.40 | 1 | 1 | 0.26 |
| 16XB28 | 28 | 6 | 3.56 | 0 | 0 | 0.00 |
| 16ZBS05 | 51 | 10 | 10.16 | 0 | 0 | 0.00 |
| 16ZBS07 | 178 | 21 | 11.38 | 3 | 2 | 0.19 |
| 16ZBM3 | 85 | 16 | 6.85 | 0 | 0 | 0.00 |
| 16ZBM1 | 67 | 15 | 5.82 | 1 | 1 | 0.09 |
| 16XB7 | 12 | 8 | 4.48 | 0 | 0 | 0.00 |
| 16ZBM2 | 102 | 16 | 5.93 | 2 | 2 | 0.12 |
| 16XB53 | 16 | 6 | 3.01 | 0 | 0 | 0.00 |
| 16XB21 | 56 | 10 | 5.97 | 1 | 1 | 0.11 |
| 16ZBM6 | 43 | 9 | 4.62 | 3 | 2 | 0.32 |
| 16ZBS63 | 61 | 6 | 4.96 | 0 | 0 | 0.00 |
| 16XB51 | 33 | 8 | 5.55 | 0 | 0 | 0.00 |
| 16ZBM5 | 75 | 12 | 5.36 | 0 | 0 | 0.00 |
| 16XB37 | 55 | 10 | 10.36 | 1 | 1 | 0.19 |
| 16ZBS09 | 2 | 2 | 4.08 | 0 | 0 | 0.00 |
| 16XB90 | 34 | 5 | 6.20 | 8 | 3 | 1.46 |
| 16XB97 | 41 | 5 | 5.41 | 0 | 0 | 0.00 |
| 16XB60 | 17 | 3 | 2.99 | 5 | 2 | 0.88 |
| 16XB92 | 40 | 11 | 2.32 | 3 | 1 | 0.17 |
| 16XB73 | 14 | 6 | 2.76 | 0 | 0 | 0.00 |
| 16XB70 | 18 | 5 | 2.40 | 4 | 2 | 0.53 |
| 16ZBM4 | 28 | 6 | 4.29 | 4 | 2 | 0.61 |
| 16XB83 | 27 | 6 | 3.31 | 36 | 2 | 4.42 |
| 16ZBS16 | 44 | 9 | 4.81 | 0 | 0 | 0.00 |
| 16ZBM7 | 21 | 6 | 10.38 | 0 | 0 | 0.00 |

*^G^ the order Gaiellales.*

*^R^ the order Rubrobacterales.*

*Ra^G^% the relative abundance of the order Gaiellales.*

*Ra^R^% the relative abundance of the order Rubrobacterales.*
