# Supplementary figures and images for: Diversity and Distribution of Uncultured and Cultured Gaiellales and Rubrobacterales in South China Sea Sediments
Source: Front Microbiol. 2021 Jun 16;12:657072. doi: 10.3389/fmicb.2021.657072 (PMC8248818; doi:10.3389/fmicb.2021.657072)

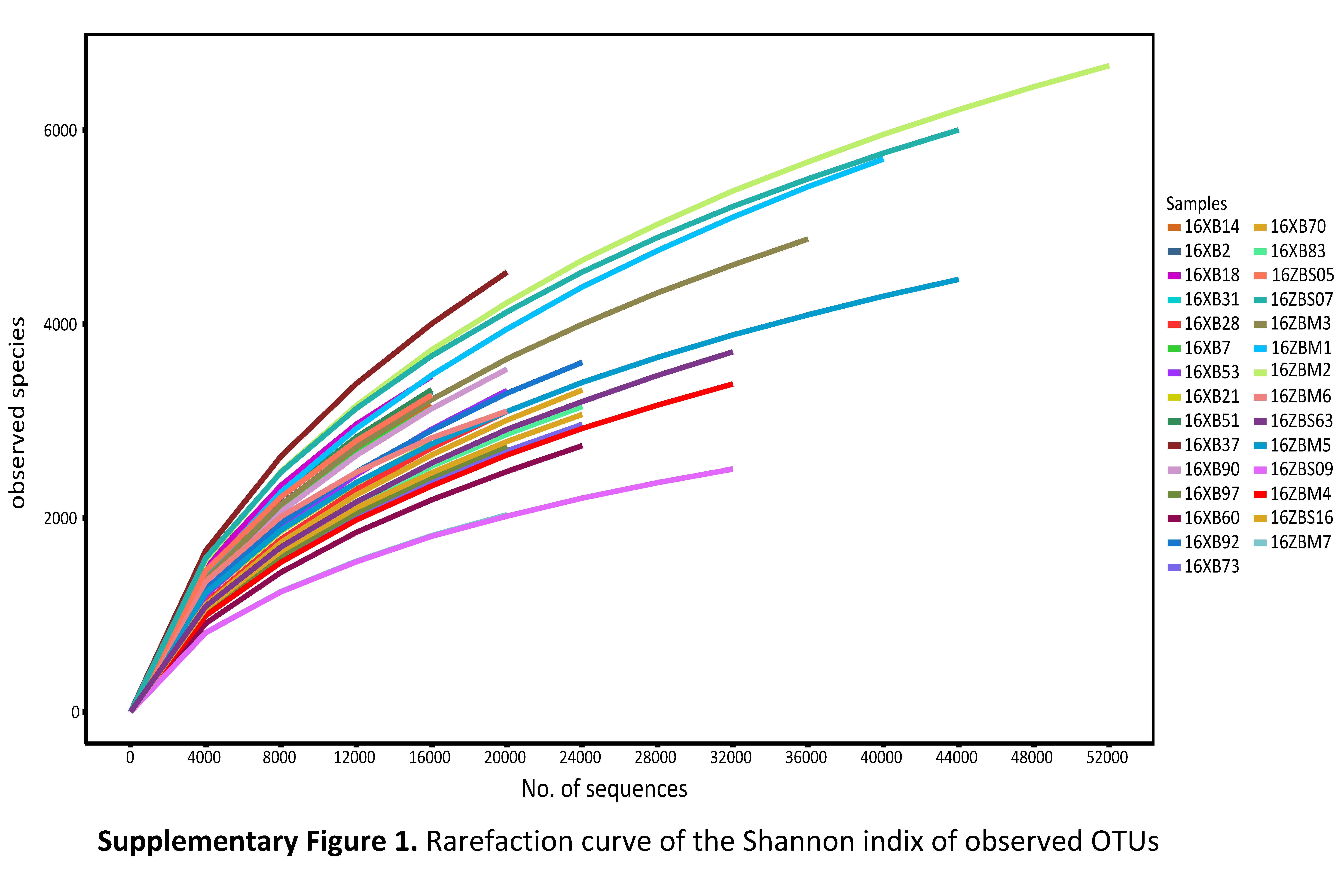

Supplement: Supplementary file 8 [file Image_1.tif]

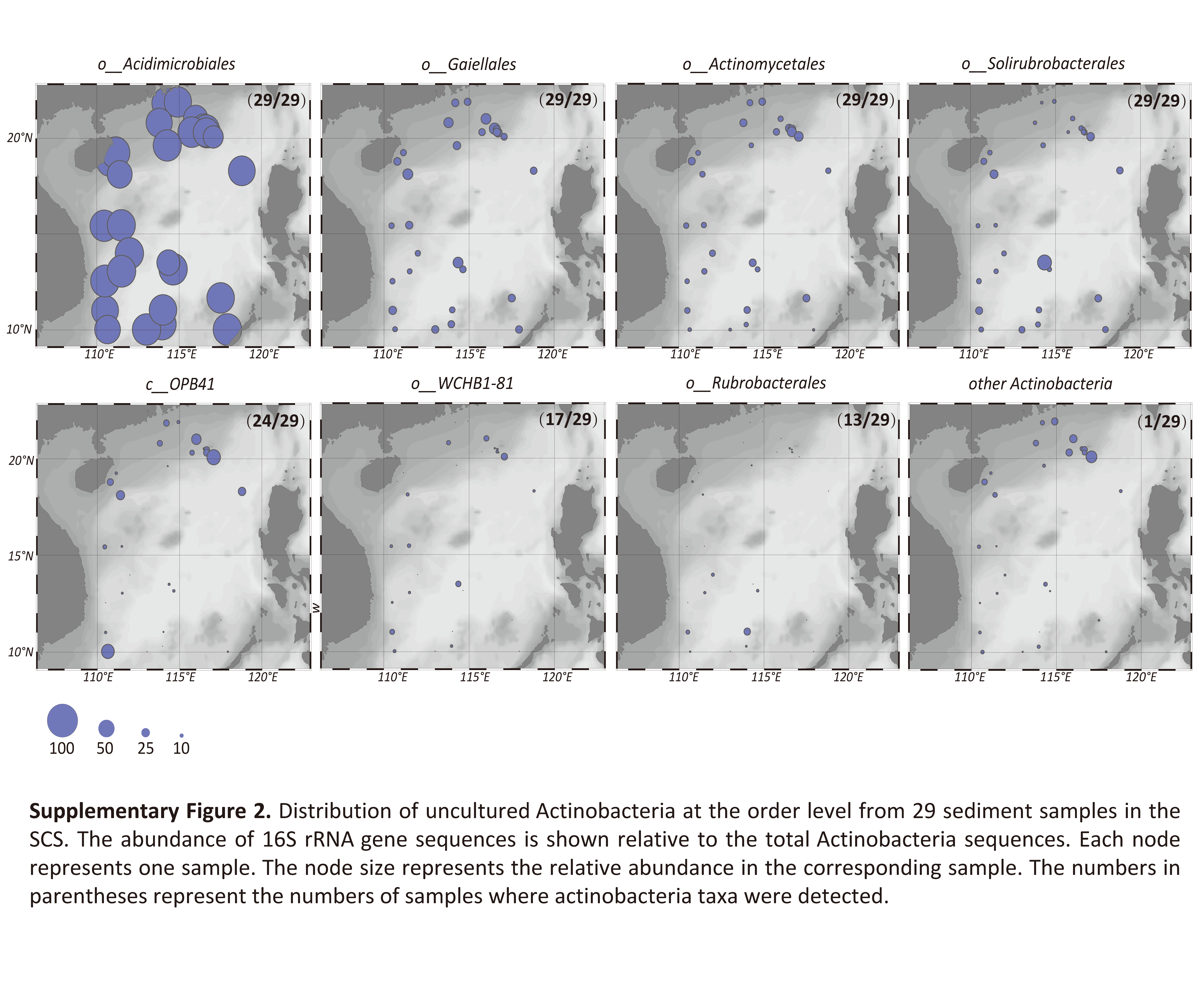

Supplement: Supplementary file 9 [file Image_2.tif]

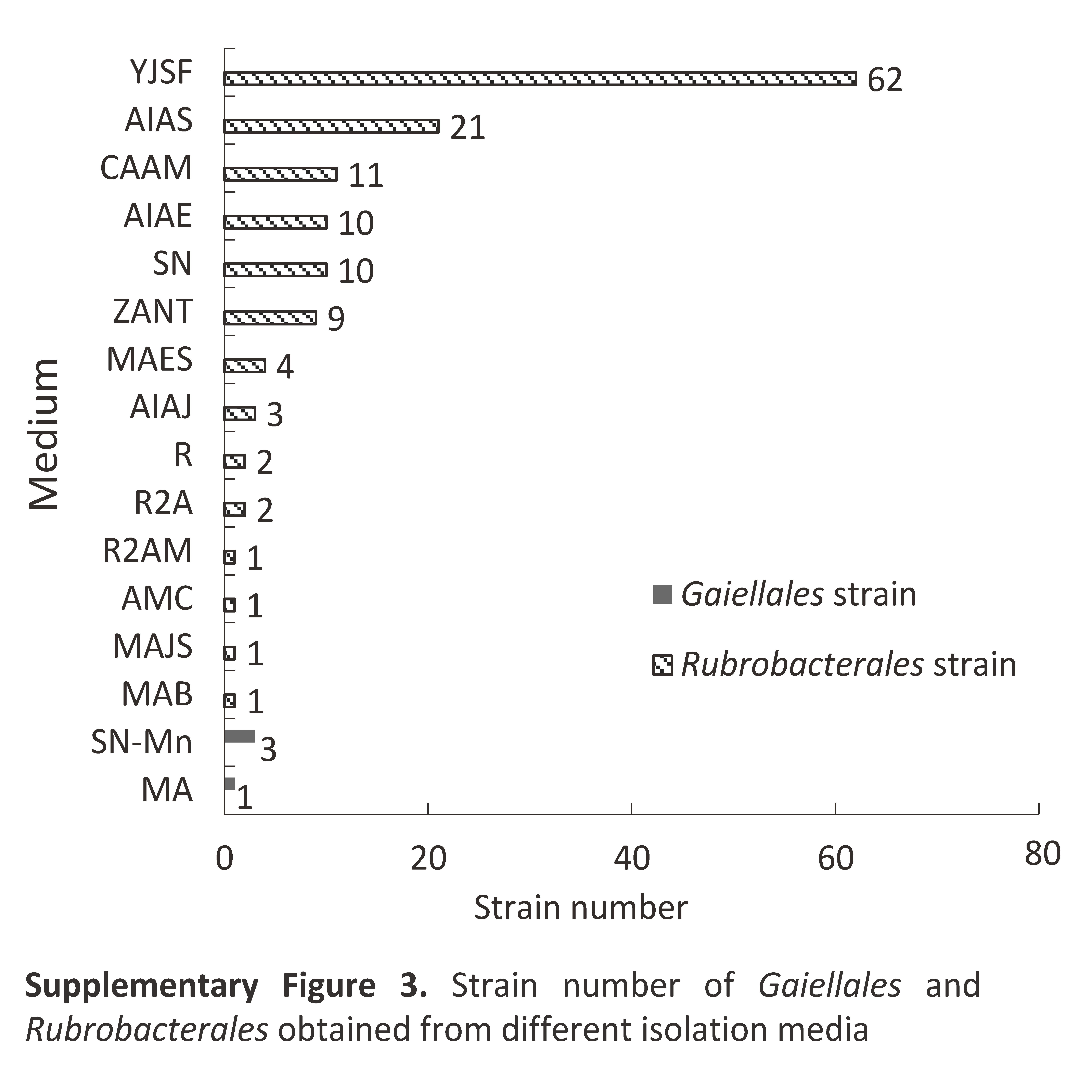

Supplement: Supplementary file 10 [file Image_3.tif]
